# Simultaneous Determination of Size and Quantification of Gold Nanoparticles by Direct Coupling Thin layer Chromatography with Catalyzed Luminol Chemiluminescence

**DOI:** 10.1038/srep24577

**Published:** 2016-04-15

**Authors:** Neng Yan, Zhenli Zhu, Dong He, Lanlan Jin, Hongtao Zheng, Shenghong Hu

**Affiliations:** 1 State Key Laboratory of Biogeology and Environmental Geology, China University of Geosciences, Wuhan, CN 430074, China; 2 Faculty of Material Science and Chemistry, China University of Geosciences, Wuhan, CN 430074, China; 3 Faculty of Earth Sciences, China University of Geosciences, Wuhan, CN 430074, China

## Abstract

The increasing use of metal-based nanoparticle products has raised concerns in particular for the aquatic environment and thus the quantification of such nanomaterials released from products should be determined to assess their environmental risks. In this study, a simple, rapid and sensitive method for the determination of size and mass concentration of gold nanoparticles (AuNPs) in aqueous suspension was established by direct coupling of thin layer chromatography (TLC) with catalyzed luminol-H_2_O_2_ chemiluminescence (CL) detection. For this purpose, a moving stage was constructed to scan the chemiluminescence signal from TLC separated AuNPs. The proposed TLC-CL method allows the quantification of differently sized AuNPs (13 nm, 41 nm and 100 nm) contained in a mixture. Various experimental parameters affecting the characterization of AuNPs, such as the concentration of H_2_O_2_, the concentration and pH of the luminol solution, and the size of the spectrometer aperture were investigated. Under optimal conditions, the detection limits for AuNP size fractions of 13 nm, 41 nm and 100 nm were 38.4 μg L^−1^, 35.9 μg L^−1^ and 39.6 μg L^−1^, with repeatabilities (RSD, n = 7) of 7.3%, 6.9% and 8.1% respectively for 10 mg L^−1^ samples. The proposed method was successfully applied to the characterization of AuNP size and concentration in aqueous test samples.

Nanoparticles (NPs), due to their special properties, have been widely used in the fields of industry, medicine, and materials. Gold nanoparticles (AuNPs), in particular, have been extensively used in catalysis^1^,^2^,^3^, as optical microscope probes^4^, biosensors^5^,^6^, targeted drug delivery^7^,^8^, and so on due to their virtues of higher electron density, dielectric properties, and catalytic effect as well as good biocompatibility^9^. However, the extensive use of NPs and their consequent release may create harmful risks to the environment and organisms^10^,^11^. Several studies have demonstrated that concentration and size greatly affect their toxicity^12^. Therefore, it is of considerable interest to develop new methods for the size characterization and quantification of gold nanoparticles^13^.

Various analytical methods have been proposed for the isolation and detection of gold nanoparticles in environment materials, including cloud point extraction (CPE)^14^,^15^,^16^, solid phase extraction (SPE)^17^,^18^, field-flow fractionation (FFF)^19^,^20^,^21^, size-exclusion chromatography (SEC)^22^,^23^, liquid chromatography (LC)^24^,^25^, hydrodynamic chromatography (HDC)^26^,^27^, capillary electrophoresis (CE)^28^,^29^,^30^ and single-particle ICP-MS (SP-ICP-MS)^31^,^32^,^33^. However, most of these methods are non-trivial and require complex and costly instrumentation^25^,^34^. To overcome these disadvantages, development of simple and cost-effective approaches for separating and quantifying nanomaterials is highly desirable. In a recent study^35^ by the authors it was demonstrated that relatively simple and inexpensive thin layer chromatography (TLC) can be successfully applied to the separation of gold nanoparticles in aqueous media. By coupling TLC with laser ablation inductively coupled plasma mass spectrometry (LA-ICP-MS), quantitative characterization of differently sized gold nanoparticles is achievable. However, the use of expensive and complex LA-ICP-MS restricts its adoption, since LA-ICP-MS is not available within most of laboratories. Therefore, it is considered important to develop a detection method which is sensitive, cheap and convenient for the study of engineered nanomaterials in environmental samples^34^.

Chemiluminescence (CL) has been established as a valuable detection technique offering low detection limits, wide linear range, high analytical throughput and simple instrumentation^36^,^37^. Tsogas *et al.* recently reported a method for the ultratrace determination of silver, gold, and iron oxide nanoparticles involving chemiluminescence detection^34^. However, the metal nanoparticles required dissociation into their precursor metal ions prior to chemiluminescence detection, which is both tedious and time consuming^37^. In recent years, it is well reported that nanoparticles can participate in CL reactions as reductants, catalysts, and luminophors^38^,^39^. AuNPs as catalysts in CL reactions have received much attention^40^,^41^,^42^,^43^, and may catalyze the decomposition of hydrogen peroxide to produce reactive oxygen species and enhance the CL by the reaction between luminol (3-aminophthalhydrazide) and hydrogen peroxide^40^,^44^, leading to its wide application in bioanalysis^45^,^46^ and immunoassay^47^,^48^. Although the catalytic ability of AuNPs for luminol–H_2_O_2_ CL reaction has been widely recognized, the use of CL for the characterization of differently sized AuNPs has not been reported.

In this study, we present a simple analytical methodology for the determination of differently sized gold nanoparticles by direct coupling of thin layer chromatography with catalyzed luminol-H_2_O_2_ chemiluminescence. Gold nanoparticles, which represent one of the most widely utilized nanoparticles, were separated by thin layer chromatography. The HPTLC plate containing the separated particles was sprayed with luminol and peroxide, the chemiluminescence signals then being monitored along the TLC track. In this manner, the separated differently sized AuNPs could be characterized and quantified within 45 s. Experimental values of concentration of H_2_O_2_, the concentration of luminol and size of the spectrometer aperture were adjusted to allow sensitive and reproducible detection of different sizes of AuNPs.

## Result and Discussion

### Direct determination of AuNPs by catalyzed Luminol–H_2_O_2_ CL reaction

It has been reported that in alkaline media, the oxidation of luminol by H_2_O_2_ generates CL^49^ and gold nanoparticles significantly enhance the luminol-H_2_O_2_ aqueous solution CL signals^40^. The coupling of CL with TLC for the analysis of nanoparticles has not been reported, so the catalytic performance of gold nanoparticles on HPTLC plates on luminol-H_2_O_2_ chemiluminescence was firstly investigated. AuNPs with 13, 41 and 100 nm diameters were deposited onto HPTLC plates. After air drying, the HPTLC plate was secured on a translation stage using double-sided adhesive tape and placed into a sampling chamber firstly, and then the chemiluminescence signals were monitored immediately after the HPTLC plate was sprayed with solutions of H_2_O_2_ and luminol in turn using a TLC sprayer.

**Figure 1:**
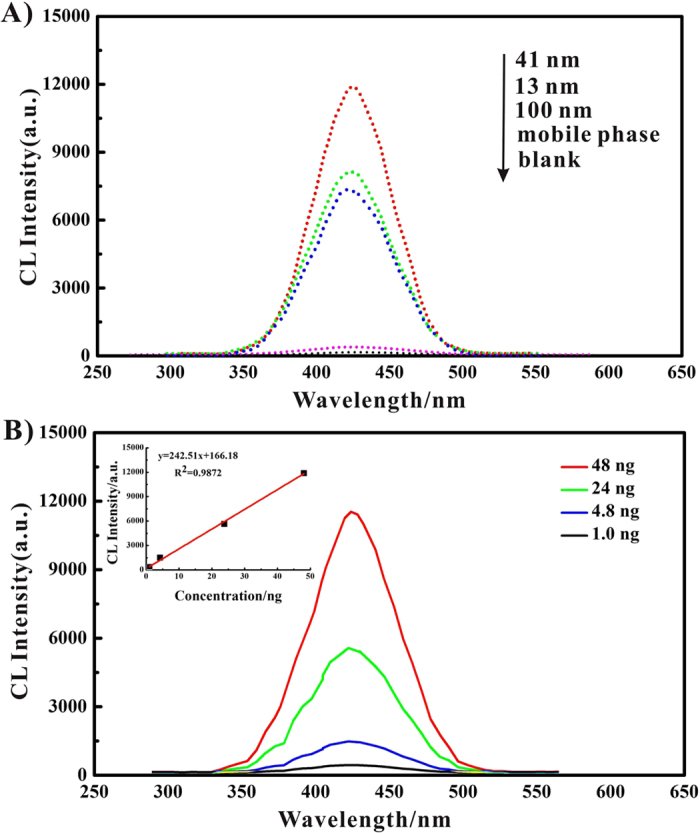
(**A**) Chemiluminescence spectra for luminol-H_2_O_2_ from gold nanoparticles on the HPTLC plate. (**B**) Concentration-dependent chemiluminescence profiles spectra of AuNPs (illustrated with 41 nm particles). Condition: pH: 12, luminol concentration: 0.8 mmol L^−1^, H_2_O_2_ concentration: 1.0 mol L^−1^.

As shown in Figure 1A, it was observed that in the absence of gold nanoparticles, no obvious CL emission was observed for the luminol–H_2_O_2_ system. However, in the presence of gold nanoparticles, intense CL signals were detected, which confirms that gold nanoparticle enhance CL signals from the HPTLC plate. Figure 1A also suggests that the catalytic effects vary substantially with particle size with 41 nm size particles producing the best CL enhancement, which agrees with previously reported results for particles of 38 nm^40^. It was also noted that the maximum emission for all the cases occurred at a wavelength of ~425 nm. These observations are in agreement with those obtained by Zhang *et al.* who investigated the effects of gold nanoparticles on luminol-H_2_O_2_ chemiluminescent in the aqueous media^40^. It has been observed that the presence of surfactants above a critical micelle concentration enhances the CL intensity of luminol-H_2_O_2_^50^, while the presence of EDTA operates to greatly reduce the luminescence of luminol-H_2_O_2_ because of formation of metal-EDTA complexes^51^. In light of these observations it was considered important to assess the influence of the TLC mobile phase, which consists of both surfactants and EDTA, on the AuNPs CL intensity. The results indicated that the presence of the mobile phase has negligible effect on the AuNPs CL signal intensity and it is therefore possible to utilize the catalytic property of gold nanoparticles on CL to identify and quantify their existence. As shown in Figure 1B, a positive correlation exists between CL signal intensity and AuNP concentration in the tested range. These results indicate that using the present experimental setup, rapid and convenient measurements of CL emission is possible and the proposed method could be used to detect and identify AuNPs by coupling TLC with chemiluminescence. Compared with ICP-MS based methods for the determination of gold nanoparticles, the proposed method is more convenient, low-cost and fast, and allows determination of different sizes of gold nanoparticles in a single run.

### Effectiveness of TLC-CL for AuNP size determination

**Figure 2:**
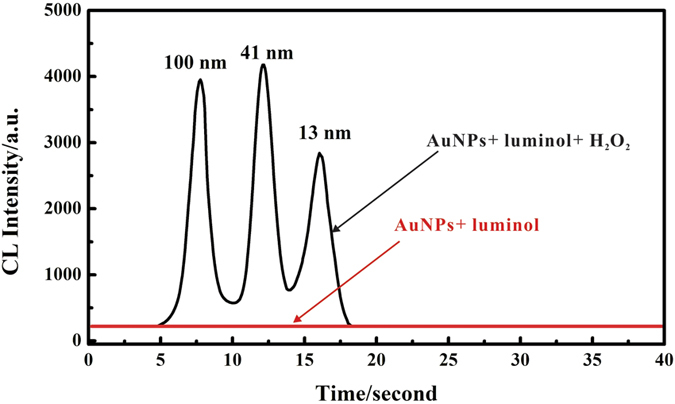
Ability of TLC-CL method to detect and discriminate differently sized AuNPs. Condition: pH: 12, luminol concentration: 0.8 mmol L^−1^, H_2_O_2_ concentration: 1.0 mol L^−1^. Scanning rate: 3.0 mm s^−1^.

In a previous study by the authors^35^, it has been demonstrated that TLC could effectively separate different sizes of AuNPs and smaller particles migrated faster than larger ones. In order to investigate the capability of TLC-CL method in size determination of AuNPs, a mixture of 13, 41, and 100 nm AuNPs was deposited onto HPTLC plates and processed as previously described. Figure 2 depicts the characteristic CL signal profile obtained by line scanning along the TLC channel. Three characteristic peaks corresponding to 13 nm, 41 nm and 100 nm AuNPs, respectively, were observed. The HPTLC plate deposited with water only (blank) and treated with luminol and H_2_O_2_ yielded a negligible CL signal. Based on these observations, it appears that by coupling TLC with CL, it is possible to separate and quantify 13 nm, 41 nm and 100 nm AuNPs in one analytical step. All results demonstrate that TLC-CL method may be applied in the size determination for AuNPs though Figure 2 does show some peak overlap indicating clean baseline separation of signals from 13 nm, 41 nm and 100 nm particle signal is not achieved. These initial results were very encouraging and further experiments were performed to improve the proposed TLC-CL method.

### Optimization of Chemiluminescence Detection

To improve the CL reaction and detection conditions, the experimental parameters affecting CL detection were systematically investigated varying one parameter at a time while keeping the rest constant.

### Detection time for Luminol-H_2_O_2_ CL reaction catalyzed by AuNPs

CL emission is a transient phenomena appearing immediately when the HPTLC plate is sprayed with luminol and peroxide, the emitted light from the reactions is also decaying with time. To guarantee both the accuracy and precision of the procedure it is therefore necessary to optimize the detection time for the CL reaction. AuNPs solutions with 13, 41 and 100 nm diameters were spotted separately onto the HPTLC plates. After air drying and treatment with luminol and H_2_O_2_ the chemiluminescence signals were monitored under light of wavelength 425 nm.

**Figure 3:**
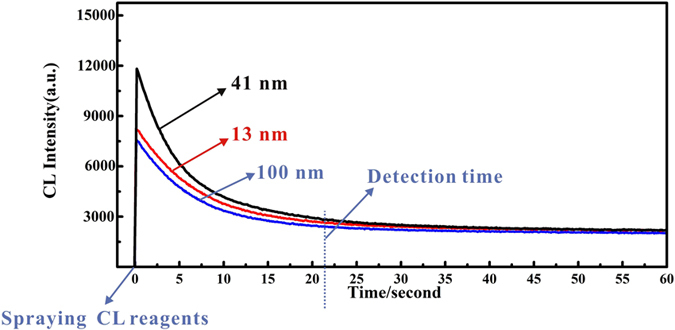
CL intensity-time curves for differently sized AuNPs. (Equal amounts of 13, 41 and 100 nm AuNPs (48 mg L^−1^) manually deposited on HPTLC plate). Condition: pH: 12, luminol concentration: 0.8 mmol L^−1^, H_2_O_2_ concentration: 1.0 mol L^−1^.

Figure 3 shows the CL emission intensity-time profiles of the samples so prepared. It can be observed that CL emission appears immediately the HPTLC plate is sprayed followed by a rapid signal decay. Due to the difference in catalytic efficiency of AuNPs on luminol-H_2_O_2_, the slope of the CL intensity-time curve for different sizes of AuNPs is different. Though the CL emission is higher for 41 nm AuNPs the signal decays more rapidly than for 13 nm and 100 nm AuNPs, indicating that higher catalytic efficiency leads to the faster reduction of the CL emission. After 20 s, the CL signal is observed to decay more slowly leveling off at 20–30% of the initial peak height, the variation of CL intensity being within 6.5% from 25 s to 60 s. It is concluded that in order to achieve best precision and reproducibility, the optimal detection time for AuNPs of a range of sizes should begin at 27 s. It should be noted that slight difference in CL intensity for differently sized AuNPs was observed for this time. Under the proposed analytical scheme the scanning time for developed TLC plates, from the scanning start point to the 100 nm AuNP position is approximately 7 s. Therefore, after the process of securing and spraying, the HPTLC-plate the plate is exposed in atmosphere for approximately 20 s, to allow the signal to decay to our preferred start time.

### Spectrometer aperture

**Figure 4:**
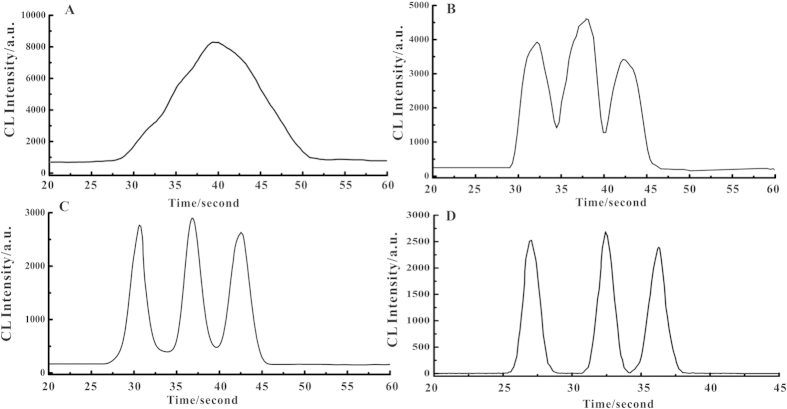
Chromatogram scans of 48 mg L^−1^ of 13 nm, 41 nm and 100 nm particles with different sizes of spectrometer scanning aperture (**A**) 1.38 cm; (**B**) 0.85 cm; (**C**) 0.61 cm; (**D**) 0.26 cm. Condition: pH: 12, luminol concentration: 0.8 mmol L^−1^, H_2_O_2_ concentration: 1.0 mol L^−1^. Scanning rate: 3.0 mm s^−1^.

While a larger optical aperture in our spectrometer will obviously increase the signal intensity, the achievable physical resolution will decrease. It is therefore necessary to optimize the size of the spectrometer aperture. Figure 4 displays sample spectra for different sizes of AuNPs separated on a single HPTLC plate obtained using apertures of size 1.38 cm, 0.85 cm, 0.61 cm and 0.26 cm respectively. Figure 4A shows that the peaks for 13 nm, 41 nm and 100 nm particles can not be distinguished when the aperture is 1.38 cm while a 0.26 cm aperture shows clear separation of the peaks, though this gain in resolution was accompanied by a loss in sensitivity. The aperture of 0.26 cm was adopted in this study since the signal loss was considered acceptable. It is also possible to improve the resolution by reducing the translation stage scan rate from 3.0 to 1.0 mm s^−1^. However, significant peak distortion was observed at this slower scan rate and so to produce best resolution with acceptable sensitivity for the detection of AuNPs a scan speed of 3.0 mm s^−1^ was selected.

### pH of luminol solution

**Figure 5:**
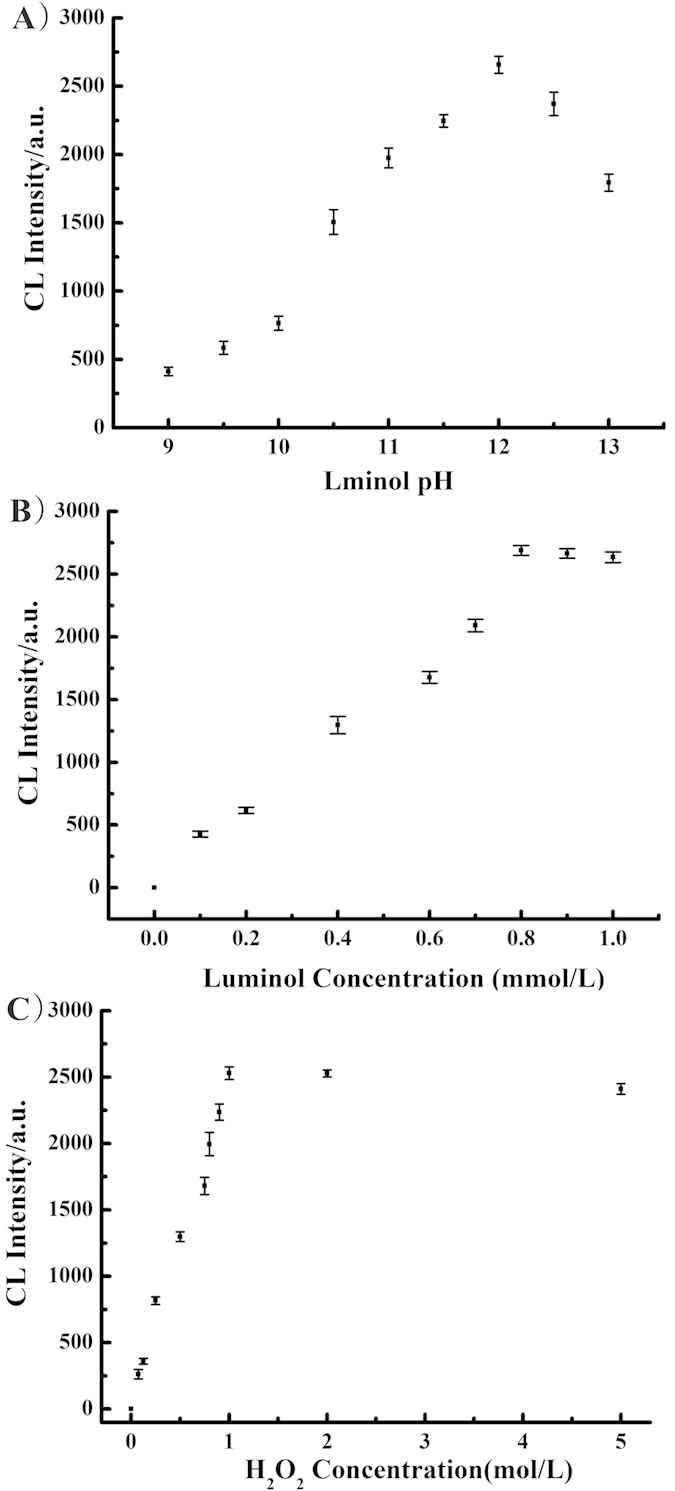
The optimization of chemiluminescence detection. (**A**) pH variation on the CL emission from 41 nm AuNPs (luminol concentration: 0.8 mmol L^−1^, H_2_O_2_ concentration: 1.0 mol L^−1^). (**B**) luminol concentration versus CL emission; (H_2_O_2_ concentration: 1.0 mol L^−1^, pH: 12). (**C**) H_2_O_2_ concentration versus CL emission; (luminol concentration: 0.9 mmol L^−1^, pH: 12).

It is reported that luminol is stabilized by protonation^52^ and the luminol-H_2_O_2_ CL is optimal at an alkaline pH^53^, therefore the effect of pH on CL reaction of luminol was investigated under alkaline conditions. Luminol was dissolved in NaOH solution of varying concentrations covering a pH range of 9.0–13.0. Shown in Figure 5A, AuNPs exhibited the strongest catalytic effect and maximum CL intensity at pH 12. In the range of pH 9.0–12.0, CL emission was observed to increase with increasing pH, while CL intensity decreased in the range pH 12.0–13.0. From this result, a pH of 12 was selected for the study.

### Luminol and H_2_O_2_ concentration

Reaction kinetics suggest that the concentration of luminol and H_2_O_2_ are important in the CL reaction^40^, affecting both the reaction rate and the CL intensity. Luminol concentration was varied in the range 0–1 mmol L^−1^, Figure 5B showing the CL emission intensity increasing with luminol concentration in the range of 0–0.8 mmol L^−1^ but only changing a little when the concentration of luminol is above 0.8 mmol L^−1^.

Similarly H_2_O_2_ concentration was varied over the range 0–5.0 mol L^−1^, with CL emission increasing markedly with increasing concentration of H_2_O_2_ up to 1 mol L^−1^ but for higher concentrations the signal remained constant and even showed some decrease (Figure 5C). The observed slight decrease at higher concentrations may be a result of emission instability. Based on these results, concentrations of 0.8 mmol L^−1^ luminol and 1.0 mol L^−1^ H_2_O_2_ were selected in subsequent experiments.

### Analytical performance

**Figure 6:**
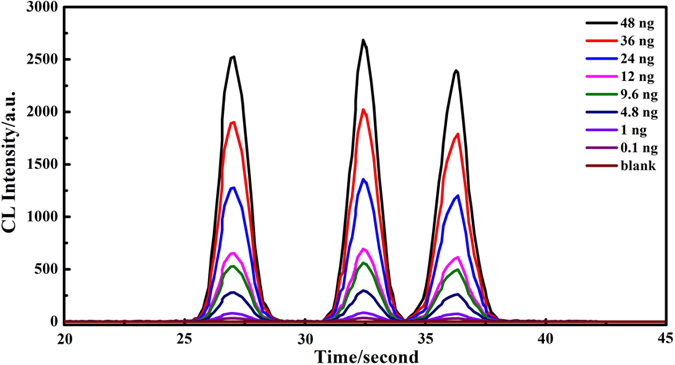
CL emission curves for 13 nm, 41 nm and 100 nm particles for varying concentration. Condition: pH: 12, luminol concentration: 0.8 mmol L^−1^, H_2_O_2_ concentration: 1.0 mol L^−1^.

**Figure 7:**
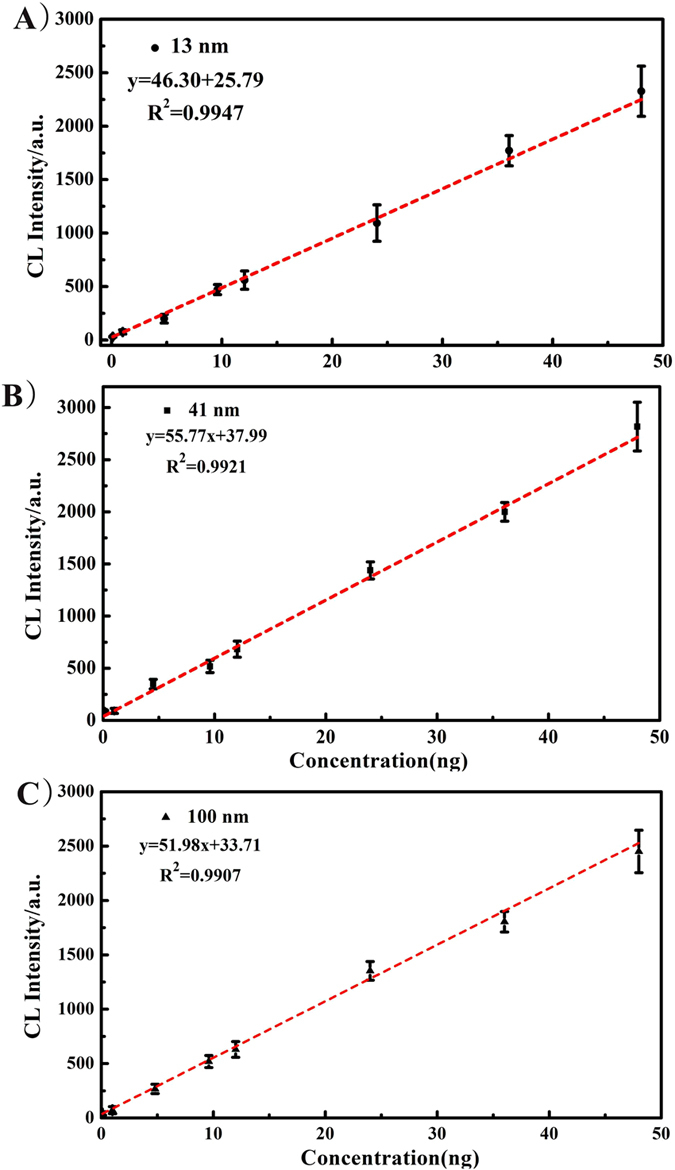
The linear relationship between CL intensity and AuNPs concentration. (**A**) 13 nm, (**B**) 41 nm, (**C**) 100 nm.

To assess the sensitivity of the developed method and determine its suitability for AuNP quantitation, we investigated CL of 13, 41 and 100 nm AuNPs over the concentration range 0.1–48 mg L^−1^. The peak height was used as the target analytical measurement throughout. Clearly evident in Figure 6 the CL signal intensity positively correlated with AuNP concentrations in the tested range. The resultant data show a linear response with correlation coefficients better than 0.99 in all studied cases (Table 1 and Figure 7). The limits of detection for 13 nm AuNPs, 41 nm AuNPs and 100 nm AuNPs, defined as three times the standard deviation of blank signal intensity (LODs, 3σ) in 11 runs, were calculated to be 38.4 μg L^−1^, 35.9 μg L^−1^ and 39.6 μg L^−1^, respectively. Therefore, in our proposed study, it is possible to quantitative characterize of AuNPs mixtures of various sizes in the 13–100 nm range which is comparable with the chromatographic methods such as field flow fractionation (FFF) (separation of AuNP mixture of around 8 nm, 20 nm, and 45 nm^19^). Moreover, the limits of detection of the proposed method for 13 nm AuNPs, 41 nm AuNPs and 100 nm AuNPs are calculated to be 38.4 μg L^−1^ (38.4 pg), 35.9 μg L^−1^ (35.9 pg) and 39.6 μg L^−1^ (39.6 pg), respectively, which are comparable to FFF (20 pg-400 pg)^54^,^55^ when coupled to ICP-MS. It should be noted that the LODs of the proposed method is even lower than the previous work by the authors which coupled TLC with LA-ICP-MS (87 μg L^−1^, 79 μg L^−1^, and 72 μg L^−1^ for 13, 34, and 47 nm AuNPs)^35^. In our future research, we will surely optimize the experimental setup and experiment process to improve the separation resolution and LODs of our method.

In addition, the analysis time using TLC-CL of 45 s is far superior when compared with TLC-LA-ICPMS (600 s). The relative standard deviations (RSD) for seven replicate determinations of 10 mg L^−1^ target species were 7.3%, 6.9% and 8.1% for 13 nm AuNPs, 41 nm AuNPs and 100 nm AuNPs, respectively. All these results demonstrate that the proposed method can provide a fast, sensitive and cost-effective method for the quantitative characterization of AuNPs.

### Interference from coexisting ions and dissolved organic matters (DOM)

The potential applications of chemiluminescence (CL) in analytical chemistry take advantage of the high sensitivity and simplicity of instrumentation associated with CL-based detection. However, lack of chemical selectivity is a practical problem which may be encountered in CL analysis. Likely coexisting metal ions and DOM that might react with the CL reactant were examined for their effect on the recovery of different sizes of AuNPs. Details of the effect of coexisting ions and humic acids are summarized in Table 2 and Figures S1 (see details in Supplementary Information). The experimental results show that the recovery of AuNPs in the simulated water samples is in the range of 92.5–102.4%, indicating that coexisting ions have negligible effects on the method. It has been reported that DOM such as humic acid can associate with various NMs to form stable suspensions therefore it was considered prudent to check if the presence of humic acid, widely present in environmental water, may influence the separation and detection of AuNPs. To simulate the effect of DOM on the extraction process, commercially available humic acid (HA) was used in different concentrations with different sizes of AuNPs, the HA stock suspension (10 g L^−1^) being diluted to produce samples with HA in an environmentally relevant concentration range from 0 to 30 mg L^−1^. It was observed that the recovery remained constant for HA concentrations up to 10 mg L^−1^, and higher than 85% when the concentration of humic acid was below 20 mg L^−1^. Only for HA levels higher than 20 mg L^−1^ was significantly lower recovery observed (Figure S1, Supplementary Information). Since in most waters, especially fresh waters, the HA concentrations are typically below 20 mg L^−1^, the effect of DOM on AuNP measurement may be regarded as not being significant.

The assessment of interference by likely coexisting metal ions and humic acid in environmental waters suggest analysis of citrate stabilized AuNPs will not be affected. Coupling chemiluminescence with TLC separation has successfully overcome the selectivity of chemiluminescence and has improved chemical specificity in analytical measurements of a complex nature.

Since the catalytic effect of gold nanoparticles on Luminol was first reported by Cui *et al.*^40^, a great variety of nanoparticles (NPs) have been found to possess similar catalytic properties, e.g., triangular AuNPs^42^, AgNPs^56^, PtNPs^57^, quantum dots^58^, and so on^37^. Therefore, the interference from AgNPs has been investigated in our experiment. The recovery of 41 nm AuNPs is approximately 85%, indicating that the AgNPs has no significant effects on the method. In our future research, we will investigate interference from different kinds of nanoparticles to make our proposed method much more practical and can be used in the field of environmental analysis.

### Application to Real Water Samples

According to our study, smaller particles (13 nm) migrate faster than larger ones (100 nm) and a good linear correlation between the particle size and the migration distance is obtained (R^2^ = 0.9918)^35^. Moreover, the CL intensity is linearly correlated with the concentration of AuNPs in our proposed study. Thus, we can obtain quantitative results and the size information of gold nanoparticles base on the migration distance and CL signal of gold nanoparticles perform in the experiment. The feasibility of the proposed approach was evaluated by measurement of three real water samples, tap, river and lake, spiked with AuNPs in a concentration range from 0 to 10 mg L^−1^. All samples were measured after passing through a 0.45 μm filter to eliminate naturally occurring particles might interfere with the procedure. Table 3 summarizes the AuNP recoveries obtained for the three real water samples. The recovery for 13, 41, and 100 nm AuNPs in the three spiked water samples was in the range of 75.0–96.5%, 78.0–103.0%, and 79.0–98.5%, indicating that the proposed method is capable of analyzing AuNPs in this concentration range in natural water samples.

## Conclusion

The direct coupling of thin layer chromatography with chemiluminescence detection applied to the separation and quantitative characterization of differently sized AuNPs was demonstrated. Detection limits for AuNPs analyzed by the developed technique were at the pg level and the recovery from real waters confirms the feasibility of this approach. Compared with established methods, the proposed method has the advantages of simplicity, high sensitivity, convenience, fast operation and requires no complex instrument. It provides an alternative way for the quantification of AuNPs. Further work need to be performed to extend to other metal based NPs.

## Methods

### Instrumentation

**Figure 8:**
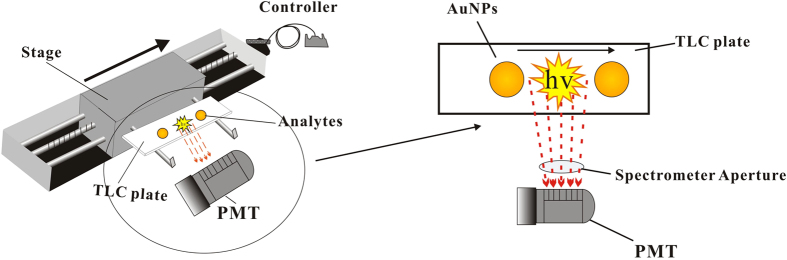
Schematic diagram of the TLC-CL experimental setup.

Figure 8 shows a schematic diagram of the experimental setup of the TLC-CL apparatus. Chromatography was performed on silica gel 60 F254 HPTLC plates (Merck, Darmstadt, Germany). The sample solution containing the AuNPs was spotted (1 μL) on the HPTLC plate and developed in a mobile phase for 20 min. After development and air drying, the cut HPTLC plate (50 mm × 25 mm, L × W) was secured on a translation stage by double-sided adhesive tape before spraying with solutions of luminol, and H_2_O_2_ using a thin-layer chromatograph sprayer, and then placed in a sampling chamber. The translation stage was driven by a motor moving the sample at a rate of between 1.0 and 4.0 mm s^−1^. The light output from the catalyzed CL reaction was detected by a Model GD-1 luminometer (Ruimai Electronic Science Co., China) which was equipped with a Model CR105 photomultiplier tube (PMT) (Bingsong Electronic Co., Beijing, China). The transmission electron micrograph (TEM) images of the AuNPs were captured on a JEM-2010 electron microscope (Philips CM12 TEM/STEM, Netherlands) and recorded using a UV-vis spectrophotometer-1750 (Shimadzu, Japan).

### Reagents

All reagents used were at least of analytical agent grade and 18.2 MΩ cm^−1^ resistivity water (90005-02, Labconco Water Pro Ps, Canada) was used throughout this study. A 2.5 × 10^−2^ mol L^−1^ stock solution of luminol (3-aminophthalhydrazide) (Sigma, USA) was prepared by dissolving luminol in 0.1 mol L^−1^ sodium hydroxide solution. Working solutions of luminol were prepared by diluting the stock solution. Working solutions of H_2_O_2_ were prepared daily from 30% (v/v) H_2_O_2_ (Xinke Electrochemical Reagent Factory, Bengbu, China). Sodium citrate and Triton-X 114 was purchased from Sigma-Aldrich. HAuCl_4_·4H_2_O (48%, w/w) was obtained from Sinopharm Group Chemical Reagent Co. Ltd. (Shanghai, China).

### Preparation and Characterization of AuNPs

The procedure described by Frens *et al.*^59^ was adopted. 50 mL of 0.01% w/v HAuCl_4_ solution was transferred to a flask and heated to boiling. With vigorous stirring, 2.0 mL of 1.0% w/v trisodium citrate solution was quickly added. The color of the solution changed from pale yellow to wine red in a few seconds. The solution was refluxed for 30 min. After cooling down, the AuNP solution was kept at 4 °C in the refrigerator. The size and monodispersity of AuNPs thus prepared were determined using a transmission electron microscope and UV-VIS spectroscopy. AuNPs of differing sizes, i.e. 40 and 100 nm, were prepared by changing the volume of trisodium citrate solution added to the HAuCl_4_ solution. Figure S2 in the Supplementary Information section shows the result of TEM and UV-VIS of the self-prepared AuNPs with sizes of ~13, ~41 and ~100 nm.

### Procedure for quantitative characterization of gold nanoparticles by TLC-CL

The experimental procedure for TLC development was similar to that described in the authors’ previous work^35^. The sample solutions containing the differently sized AuNPs were spotted (1 μL) onto the HPTLC plates and developed in a mobile phase containing 0.2 M phosphate buffer (pH = 6.8), Triton X-114 (0.4%, w/v), EDTA (10 mM) for 20 min. After development and air drying, the HPTLC plate was secured on a stage using double-sided adhesive tape and placed into a sampling chamber, and then the HPTLC plate was sprayed first with a solution of H_2_O_2_ and then luminol using a TLC sprayer. The chemiluminescence signal is transitory and the CL intensity will decaying along with time, in order to guarantee both the accuracy and precision, the above procedure must be carried out at optimal time. In our experiment, after the process of securing and spraying the HPTLC-plate, the HPTLC plate then exposed to atmosphere for approximately 20 s, and finally the chemiluminescence signals were monitored along the TLC track at an optimum scan speed of 3.0 mm s^−1^. The starting position for scanning is approximately 2.0 cm away from 100 nm AuNPs, thus moving from the starting positions to 100 nm AuNPs location takes less than 10 s.

## Additional Information

**How to cite this article**: Yan, N. *et al.* Simultaneous Determination of Size and Quantification of Gold Nanoparticles by Direct Coupling Thin layer Chromatography with Catalyzed Luminol Chemiluminescence. *Sci. Rep.* **6**, 24577; doi: 10.1038/srep24577 (2016).

## Supplementary Material

Supplementary Information

## Figures and Tables

**Table 1:** The analytical performance of the proposed TLC-CL method.

Target analytes	Linear equation	R	Linear range (mg L^−1^)	LOD (pg)	RSD (%, n = 5)
13 nm AuNPs	y = 46.53x + 26.01	0.9947	0.05–60	38.4	7.3
41 nm AuNPs	y = 55.77x + 37.99	0.9921	0.05–60	35.9	6.9
100 nm AuNPs	y = 51.98x + 33.71	0.9907	0.05–60	39.6	8.1

y: Au signal intensity, x : concentration of the sample.

**Table 2:** Effect of coexisting metal ions.

Coexisting substance	Concentration (mg L^−1^)	Recovery (%)		
		13 nm	41 nm	100 nm
K^+^(Cl^−^)	1.6	97.4	98.3	97.5
Na^+^(Cl^−^)	10.0	93.9	94.9	92.5
Mg^2+^(SO_4_^2−^)	30	93.6	94.4	92.5
Ca^2+^(Cl^−^)	85	92.8	95.7	94.7
Fe^2+^(Cl^−^)	5.0	100.2	101.9	101.5
Ni^2+^(Cl^−^)	5.0	101.3	102.4	102.2
Cu^2+^(Cl^−^)	10.0	98.8	97.4	97.5
Co^3+^(Cl^−^)	5.0	98.2	100.8	98.6

**Table 3:** Analytical results of 13 nm, 41 nm and 100 nm AuNPs spiked in water samples.

Sample	Spiked (mg L^−1^)	Found (mg L^−1^)			Recovery (%)		
		13 nm	41 nm	100 nm	13 nm	41 nm	100 nm
East Lake water	0	N.D.^a^	N.D.^a^	N.D.^a^	—	—	—
	1	0.75	0.78	0.79	75.0	78.0	79.0
	10	8.74	8.58	8.65	87.4	85.8	86.5
Yangtze River water	0	N.D.^a^	N.D.^a^	N.D.^a^	—	—	—
	1	0.83	0.84	0.83	83.0	84.0	83.0
	10	8.84	8.71	8.64	88.4	87.1	86.4
Tap Water	0	N.D.^a^	N.D.^a^	N.D.^a^	—	—	—
	1	0.92	0.93	0.92	92.0	93.0	92.0
	10	9.65	10.3	9.85	96.5	103.0	98.5

^a^Below the limit of detection.

## References

[b1] Chen X., Zhu H. Y., Zhao J. C., Zheng Z. F. & Gao X. P. Visible-Light-Driven Oxidation of Organic Contaminants in Air with Gold Nanoparticle Catalysts on Oxide Supports. Angew. Chem. 120, 5433–5436 (2008). 10.1002/anie.200800602 18548470

[b2] Daniel M.-C. & Astruc D. Gold nanoparticles: assembly, supramolecular chemistry, quantum-size-related properties, and applications toward biology, catalysis, and nanotechnology. Chem. Rev. 104, 293–346 (2004). 14719978 10.1021/cr030698+

[b3] La T. A. *et al.* Assembly, Growth, and Catalytic Activity of Gold Nanoparticles in Hollow Carbon Nanofibers. Acs Nano 6, 2000–2007 (2012). 22356571 10.1021/nn300400z

[b4] Kempen P. J. *et al.* A correlative optical microscopy and scanning electron microscopy approach to locating nanoparticles in brain tumors. Micron 68, 70–76 (2015). 25464144 10.1016/j.micron.2014.09.004 PMC4262686

[b5] Luo P., Liu Y., Xia Y., Xu H. & Xie G. Aptamer biosensor for sensitive detection of toxin A of Clostridium difficile using gold nanoparticles synthesized by Bacillus stearothermophilus. Biosens. Bioelectron. 54, 217–221 (2014). 24287407 10.1016/j.bios.2013.11.013

[b6] Lepinay S., Staff A., Ianoul A. & Albert J. Improved detection limits of protein optical fiber biosensors coated with gold nanoparticles. Biosens. Bioelectron. 52, 337–344 (2014). 24080213 10.1016/j.bios.2013.08.058

[b7] Kanapathipillai M., Brock A. & Ingber D. E. Nanoparticle targeting of anti-cancer drugs that alter intracellular signaling or influence the tumor microenvironment. Adv. Drug Delivery Rev. 79, 107–118 (2014). 10.1016/j.addr.2014.05.005 24819216

[b8] Austin L. A., Mackey M. A., Dreaden E. C. & El-Sayed M. A. The optical, photothermal, and facile surface chemical properties of gold and silver nanoparticles in biodiagnostics, therapy, and drug delivery. Arch. Toxicol. 88, 1391–1417 (2014). 24894431 10.1007/s00204-014-1245-3 PMC4136654

[b9] Zhang L., Chen B., He M., Liu X. & Hu B. Hydrophilic polymer monolithic capillary microextraction online coupled to ICPMS for the determination of carboxyl group-containing gold nanoparticles in environmental waters. Anal. Chem. 87, 1789–1796 (2015). 25572871 10.1021/ac503798c

[b10] Franze B. & Engelhard C. Fast separation, characterization, and speciation of gold and silver nanoparticles and their ionic counterparts with micellar electrokinetic chromatography coupled to ICP-MS. Anal. Chem. 86, 5713–5720 (2014). 24854891 10.1021/ac403998e

[b11] Soenen S. J. *et al.* Cytotoxic Effects of Gold Nanoparticles: A Multiparametric Study. Acs Nano 6, 5767–5783 (2012). 22659047 10.1021/nn301714n

[b12] Zhang S., Han G., Xing Z., Zhang S. & Zhang X. Multiplex DNA Assay Based on Nanoparticle Probes by Single Particle Inductively Coupled Plasma Mass Spectrometry. Anal. Chem. 86, 3541–3547 (2014). 24579812 10.1021/ac404245z

[b13] López-Serrano A., Olivas R. M., Landaluze J. S. & Cámara C. Nanoparticles: a global vision. Characterization, separation, and quantification methods. Potential environmental and health impact. Anal. Methods 6, 38–56 (2014).

[b14] Liu J. F., Liu R., Yin Y. G. & Jiang G. B. Triton X-114 based cloud point extraction: a thermoreversible approach for separation/concentration and dispersion of nanomaterials in the aqueous phase. Chem. Commun. 12, 1514–1516 (2009). 10.1039/b821124h 19277374

[b15] Hartmann G. & Schuster M. Species selective preconcentration and quantification of gold nanoparticles using cloud point extraction and electrothermal atomic absorption spectrometry. Anal. Chim. Acta 761, 27–33 (2013). 23312311 10.1016/j.aca.2012.11.050

[b16] Yin Y., Liu J. & Jiang G. Sunlight-induced reduction of ionic Ag and Au to metallic nanoparticles by dissolved organic matter. Acs Nano 6, 7910–7919 (2012). 22816495 10.1021/nn302293r

[b17] Su S., Chen B., He M., Xiao Z. & Hu B. A novel strategy for sequential analysis of gold nanoparticles and gold ions in water samples by combining magnetic solid phase extraction with inductively coupled plasma mass spectrometry. J. Anal. At. Spectrom. 29, 444–453 (2014).

[b18] Li L. & Leopold K. Ligand-assisted extraction for separation and preconcentration of gold nanoparticles from waters. Anal. Chem. 84, 4340–4349 (2012). 22494142 10.1021/ac2034437

[b19] Schmidt B. *et al.* Quantitative characterization of gold nanoparticles by field-flow fractionation coupled online with light scattering detection and inductively coupled plasma mass spectrometry. Anal. Chem. 83, 2461–2468 (2011). 21355549 10.1021/ac102545e

[b20] Calzolai L., Gilliland D., Garcia C. P. & Rossi F. Separation and characterization of gold nanoparticle mixtures by flow-field-flow fractionation. J. Chromatogr. A 1218, 4234–4239 (2011). 21288528 10.1016/j.chroma.2011.01.017

[b21] Ashby J., Schachermeyer S., Pan S. & Zhong W. Dissociation-based screening of nanoparticle-protein interaction via flow field-flow fractionation. Anal. Chem. 85, 7494–7501 (2013). 23859073 10.1021/ac401485j PMC3815437

[b22] Guor-Tzo Wei F.-K. L. Separation of nanometer gold particles by size exclusion chromatography. J. Chromatogr. A 836,253–260 (1999). 10.1021/ac990044u 21662743

[b23] Wei G. T., Liu F. K. & Wang C. R. Shape separation of nanometer gold particles by size-exclusion chromatography. Anal. Chem. 71, 2085–2091 (1999). 21662743 10.1021/ac990044u

[b24] Siswoyo Lim, L. W. & T T. Separation of gold nanoparticles with a monolithic silica capillary column in liquid chromatography. Anal. Sci. 28, 107–113 (2012). 22327001 10.2116/analsci.28.107

[b25] Soto-Alvaredo J., Montes-Bayon M. & Bettmer J. Speciation of silver nanoparticles and silver(I) by reversed-phase liquid chromatography coupled to ICPMS. Anal. Chem. 85, 1316–1321 (2013). 23305255 10.1021/ac302851d

[b26] Gray E. P. *et al.* Analysis of gold nanoparticle mixtures: a comparison of hydrodynamic chromatography (HDC) and asymmetrical flow field-flow fractionation (AF4) coupled to ICP-MS. J. Anal. At. Spectrom 27, 1532–1539 (2012).

[b27] Pergantis S. A., Jones-Lepp T. L. & Heithmar E. M. Hydrodynamic chromatography online with single particle-inductively coupled plasma mass spectrometry for ultratrace detection of metal-containing nanoparticles. Anal. Chem. 84, 6454–6462 (2012). 22804728 10.1021/ac300302j

[b28] Liu F.-K., Lin Y.-Y. & Wu C.-H. Highly efficient approach for characterizing nanometer-sized gold particles by capillary electrophoresis. Anal. Chim. Acta 528, 249–254 (2005).

[b29] Liu F.-K. & Wei G.-T. Adding sodium dodecylsulfate to the running electrolyte enhances the separation of gold nanoparticles by capillary electrophoresis. Anal. Chim. Acta 510, 77–83 (2004).

[b30] Ivanov M. R., Bednar H. R. & Haes A. J. Investigations of the Mechanism of Gold Nanoparticle Stability and Surface Functionalization in Capillary Electrophoresis. Acs Nano 3, 386–394 (2009). 19236076 10.1021/nn8005619 PMC2707777

[b31] Hadioui M., Peyrot C. & Wilkinson K. J. Improvements to single particle ICPMS by the online coupling of ion exchange resins. Anal. Chem. 86, 4668–4674 (2014). 24745850 10.1021/ac5004932

[b32] Tuoriniemi J., Cornelis G. & Hassellov M. Size discrimination and detection capabilities of single-particle ICPMS for environmental analysis of silver nanoparticles. Anal. Chem. 84, 3965–3972 (2012). 22483433 10.1021/ac203005r

[b33] Wang W. & Tao N. Detection, counting, and imaging of single nanoparticles. Anal. Chem. 86, 2–14 (2014). 24328222 10.1021/ac403890n PMC4272604

[b34] Tsogas G. Z., Giokas D. L. & Vlessidis A. G. Ultratrace determination of silver, gold, and iron oxide nanoparticles by micelle mediated preconcentration/selective back-extraction coupled with flow injection chemiluminescence detection. Anal. Chem. 86, 3484–3492 (2014). 24576255 10.1021/ac404071v

[b35] Yan N. *et al.* Quantitative Characterization of Gold Nanoparticles by Coupling Thin Layer Chromatography with Laser Ablation Inductively Coupled Plasma Mass Spectrometry. Anal. Chem. 87, 6079–6087 (2015). 26005902 10.1021/acs.analchem.5b00612

[b36] Fujiwara T., Murayama K. & Imdadullahkumamaru T. Automated Method for the Selective Determination of Gold by On-Line Solvent Extraction and Reversed Micellar-Mediated Luminol Chemiluminescence Detection. Microchem.J. 49, 183–193 (1994).

[b37] Giokas D. L., Vlessidis A. G., Tsogas G. Z. & Evmiridis N. P. Nanoparticle-assisted chemiluminescence and its applications in analytical chemistry. TrAC Trends Anal. Chem. 29, 1113–1126 (2010).

[b38] Li S. F. *et al.* Enhanced Chemiluminescence of the Rhodamine 6G61Cerium(IV) System by Au61Ag Alloy Nanoparticles. J. Phys. Chem. C 113, 15586–15592 (2009).

[b39] Su Y., Xie Y., Hou X. & Lv Y. Recent Advances in Analytical Applications of Nanomaterials in Liquid-Phase Chemiluminescence. Appl. Spectrosc. Rev. 49, 201–232 (2014).

[b40] Zhi-Feng Z., Hua C., Chun-Ze L. & Li-Juan L. Gold nanoparticle-catalyzed luminol chemiluminescence and its analytical applications. Anal. Chem. 77, 3324–3329 (2005). 15889925 10.1021/ac050036f

[b41] Zhang Q. L., Wu L., Lv C. & Zhang X. Y. Effect of gold nanoparticle as a novel nanocatalyst on luminol–hydrazine chemiluminescence system and itsanalytical application. J. Chromatogr. A 1242, 84–91 (2012).22560706

[b42] Li Q., Liu F., Lu C. & Lin J. M. Aminothiols Sensing Based on Fluorosurfactant-Mediated Triangular Gold Nanoparticle-Catalyzed Luminol Chemiluminescence. J. Phys. Chem. C 115, 10964–10970 (2011).

[b43] Xin W., Na N., Sichun Z., Yayan W. & Xinrong Z. Rapid screening of gold catalysts by chemiluminescence-based array imaging. J. Am. Chem. Soc. 129, 6062–6063 (2007). 17458961 10.1021/ja0702768

[b44] Duan C., Cui H., Zhang Z., Liu B. & Wang G. W. Size-Dependent Inhibition and Enhancement by Gold Nanoparticles of Luminol61Ferricyanide Chemiluminescence. J. Phys. Chem. C 111, 4561–4566 (2007).

[b45] Roda A. *et al.* Bio-and chemiluminescence in bioanalysis. Fresenius’ journal of analytical chemistry 366, 752–759 (2000). 11225786 10.1007/s002160051569

[b46] Marquette C. A. & Blum L. J. Applications of the luminol chemiluminescent reaction in analytical chemistry. Anal. Bioanal.Chem. 385, 546–554 (2006). 16715276 10.1007/s00216-006-0439-9

[b47] Fan A., Lau C. & Lu J. Magnetic bead-based chemiluminescent metal immunoassay with a colloidal gold label. Anal. Chem. 77, 3238–3242 (2005). 15889914 10.1021/ac050163b

[b48] Wang Z., Hu J., Jin Y., Yao X. & Li J. *In situ* amplified chemiluminescent detection of DNA and immunoassay of IgG using special-shaped gold nanoparticles as label. Clin. Chem. 52, 1958–1961 (2006). 16858071 10.1373/clinchem.2006.071399

[b49] Li S. F., Zhang X. M., Du W. X., Ni Y. H. & Wei X. W. Chemiluminescence Reactions of a Luminol System Catalyzed by ZnO Nanoparticles. J. Phys. Chem. C 113, 1046–1051 (2008).

[b50] Liu X., Li A., Zhou B., Qiu C. & Ren H. Chemiluminescence determination of surfactant Triton X-100 in environmental water with luminol-hydrogen peroxide system. Chem. Cent. J. 3, 1–6 (2009). 19570217 10.1186/1752-153X-3-7 PMC2715391

[b51] Koronkiewicz S. & Sobczuk M. Multicommutation flow analysis with chemiluminescence detection: application to the chromium (III) determination. Environ. Biote. 4, 25–31 (2008).

[b52] XU *et al.* Theoretical Study on the Chemiluminescence Mechanisms of Luminol-DMSO-NaOH System. Acta Chim. Sinica 64, 1981–1987 (2006).

[b53] Burguera J. L. & Townshend A. Determination of manganese(II) by a chemiluminescence reaction. Talanta 28, 731–735 (1981). 18962994 10.1016/0039-9140(81)80114-2

[b54] Mitrano D. M. *et al.* Silver nanoparticle characterization using single particle ICP-MS (SP-ICP-MS) and asymmetrical flow field flow fractionation ICP-MS (AF4-ICP-MS). J. Anal. At. Spectrom 27, 1131–1142 (2012).

[b55] Anne-Lena F., Lars D., Bj?Rn M. & Ternes T. A. ICP-MS-based characterization of inorganic nanoparticles–sample preparation and off-line fractionation strategies. Anal. Bioanal. Chem. 18, 116–119 (1982). 10.1007/s00216-013-7480-2 PMC3885803 24292431

[b56] Hao C., Feng G., Rong H. & Cui D. Chemiluminescence of luminol catalyzed by silver nanoparticles. J. Colldid. Interf. Sci 315, 158–163 (2007). 10.1016/j.jcis.2007.06.052 17681516

[b57] Xu S. L. & Cui H. Luminol chemiluminescence catalysed by colloidal platinum nanoparticles. Luminescence 22, 77–87 (2007). 17089353 10.1002/bio.929

[b58] Algar W. R., Massey M. & Krull U. J. The application of quantum dots, gold nanoparticles and molecular switches to optical nucleic-acid diagnostics. TrAC Trends Anal. Chem. 28, 292–306 (2009).

[b59] Frens G. Controlled nucleation for the regulation of the particle size in monodisperse gold suspensions. Nature 241, 20–22 (1973).

