# Supplementary material for: Simultaneous Determination of Size and Quantification of Gold Nanoparticles by Direct Coupling Thin layer Chromatography with Catalyzed Luminol Chemiluminescence
Source: Sci Rep. 2016 Apr 15;6:24577. doi: 10.1038/srep24577 (PMC4832333; doi:10.1038/srep24577)
Supplement: Supplementary Information [file srep24577-s1.doc]

**Simultaneous Determination of Size and Quantification of Gold Nanoparticles by Direct Coupling Thin layer Chromatography with Catalyzed Luminol** **Chemiluminescence**

Neng Yan^1^, Zhenli Zhu^1*^, Dong He^1^, Lanlan Jin^1^, Hongtao Zheng^2^, Shenghong Hu^1, 3^

^1^State Key Laboratory of Biogeology and Environmental Geology, China University of Geosciences, Wuhan, China, CN 430074

^2^Faculty of Material Science and Chemistry, China University of Geosciences, Wuhan, China, CN 430074

^3^Faculty of Earth Sciences, China University of Geosciences, Wuhan, China, CN 430074

^*^To whom correspondence should be addressed. Phone: +86-27-6788-3452. Fax: +86-27-6788-3456. E-mail: [zhuzl03@gmail.com](mailto:zhuzl03@gmail.com), zlzhu@cug.edu.cn

Content

**Interference from dissolved organic matters (DOM).**


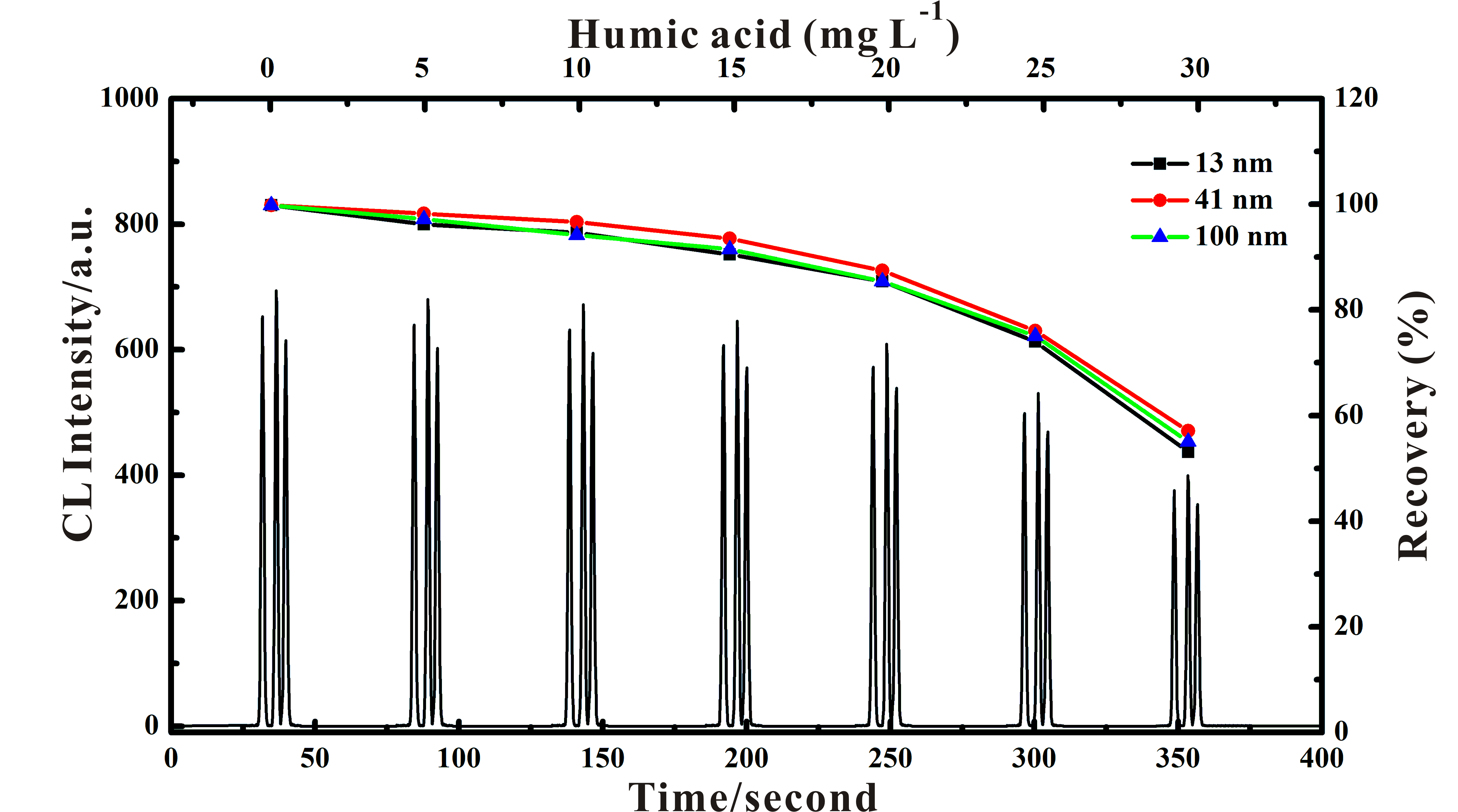


Figure S1. Effects of humic acid on the recovery of 12 ng AuNPs. pH: 12, luminol: 0.8 mmol L^-1^, H_2_O_2_: 1.0 mol L^-1^.

**Characterization of AuNPs.**


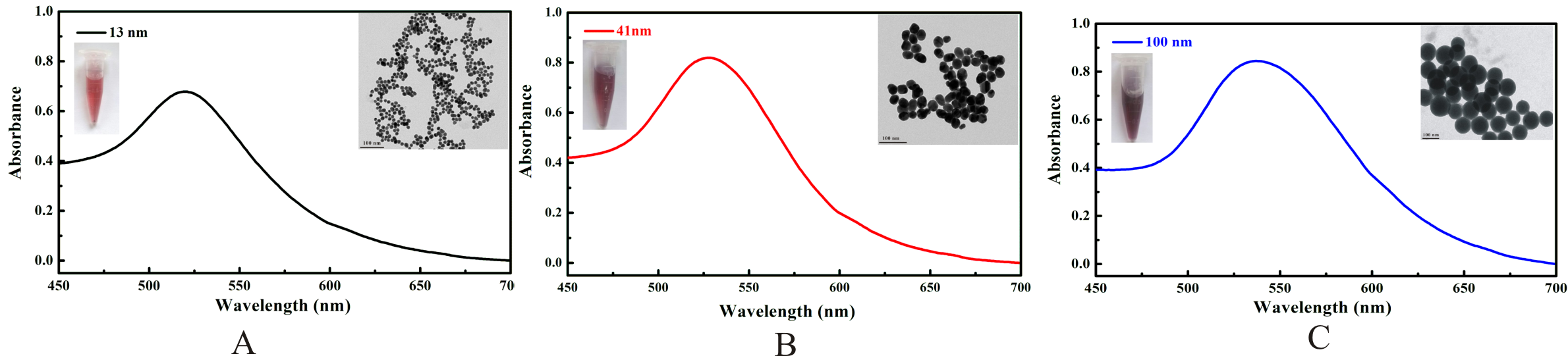


Figure S2. TEM images and UV-visible spectra of self-prepared citrate stabilized AuNPs. (A) 13 nm AuNPs, (B) 41 nm AuNPs, (C) 100 nm AuNPs.
